# Draft Genome Sequence of a Diploid and Hybrid *Candida* Strain, *Candida sanyaensis* UCD423, Isolated from Compost in Ireland

**DOI:** 10.1128/MRA.00761-21

**Published:** 2021-09-23

**Authors:** Adam Ryan, Eoin Ó Cinnéide, Sean A. Bergin, Ghozlan Alhajeri, Hawraa Almotawaa, Isabelle Daly, Sophia Heneghan, Kellie Horan, Roslyn Kavanagh, Christopher Keane, Aaron Martin, Ada McDonagh, Julia O’Leary, Matthieu Osborne, Emma Watson, Kevin P. Byrne, Kenneth H. Wolfe, Geraldine Butler

**Affiliations:** a School of Biomolecular and Biomedical Science, Conway Institute, University College Dublin, Dublin, Ireland; b School of Medicine, Conway Institute, University College Dublin, Dublin, Ireland; University of California, Riverside

## Abstract

Candida sanyaensis is a CUG-Ser1 clade yeast that is associated with soil. Assembly of short-read and long-read data shows that C. sanyaensis has a diploid and hybrid genome, with approximately 97% identity between the haplotypes. The haploid genome size is approximately 15.4 Mb.

## ANNOUNCEMENT

The yeast Candida sanyaensis is a member of the subphylum Saccharomycotina and the phylum Ascomycota and is closely related to Candida sojae and Candida tropicalis (1). The species was originally isolated from soil samples from Hainan Island in south China and Taiwan. C. sanyaensis UCD423 was isolated from a wormery in Dublin by two passages of compost material in 9 ml liquid yeast extract-peptone-dextrose (YPD) medium containing chloramphenicol (30 μg/ml) and ampicillin (100 μg/ml) and culture on YPD plates at room temperature, similar to a method reported previously (2). The species was identified from the internal transcribed spacer (ITS) sequence, which is 99% identical to that of C. sanyaensis (1).

For Illumina sequencing, total genomic DNA was extracted from a YPD culture and purified by extraction with phenol-chloroform-isoamyl alcohol. Libraries were generated from 1 μg genomic DNA and sequenced by BGI Tech Solutions Co. (Hong Kong), as described by Morio et al. (3). A total of 150 bases were sequenced from each end with an Illumina HiSeq 4000 instrument, yielding 6.5 million spots. For long-read sequencing, genomic DNA was extracted using the Qiagen Genomic-tip 100G kit. The sequencing library was generated from 400 ng of DNA using a rapid barcoding kit (SQK-RBK004) from Oxford Nanopore Technologies (ONT), following the manufacturer’s instructions. This library was mixed with three libraries from unrelated projects, purified with AMPure XP magnetic beads (Beckman Coulter), and eluted in 12 μl Tris-EDTA (TE) buffer, of which 10 μl was used for sequencing on a FLO-MIN106 flow cell primed with kit EXP-FLP002 on a MinION 1B sequencer using MinKNOW software v4.1.22 (ONT). Base calling (using the fast model [dna_r9.4.1_450bps_fast.cfg]) and demultiplexing were performed using Guppy v4.2.2 (ONT). The total number of MinION reads was 561,441, with a read *N*_50_ of 11,647 bp.

Low-quality reads (Q scores of <15) were removed from the Illumina data using Skewer v0.2.2 (4), and reads were assembled using SPAdes v3.14.0 (5) with default parameters. The MinION data were filtered using NanoFilt v2.7.1 (6), removing reads with quality scores of <10 and lengths of <10 kb. Reads were assembled using Canu v2.0 (7) with default parameters for a haploid assembly or with the options corOutCoverage = 200 and batOptions = -dg 3 -db 3 -dr 1 -ca 500 -cp 50 for a diploid assembly, assuming a genome size of 18 Mb. Assembly statistics were visualized using QUAST (8) (Table 1). Very different assemblies were obtained depending on the method and parameters used (Table 1).

**TABLE 1:** Assembly statistics for C. sanyaensis (for scaffolds of >500 bp)

Parameter	Data for assembly with:		
	SPAdes	Canu (haploid)	Canu (diploid)
No. of contigs^*a*^	9,833	144	207
Total length (bp)^*b*^	17,851,344	24,222,500	31,277,210
Size of largest contig (bp)	177,256	2,922,747	2,944,088
*N*_50_ (bp)^*b*^	2,983	1,214,081	281,063
*L*_50_	1,342	6	12
GC content (%)	31.1	31.6	31.5

a The SPAdes assembly of the short-read data alone is highly fragmented. The haploid Canu assembly of the long-read data has the smallest number of contigs.

b For the long-read data alone, the haploid assembly has a smaller genome size and a greater *N*_50_ value than the diploid assembly.

**FIG 1:**
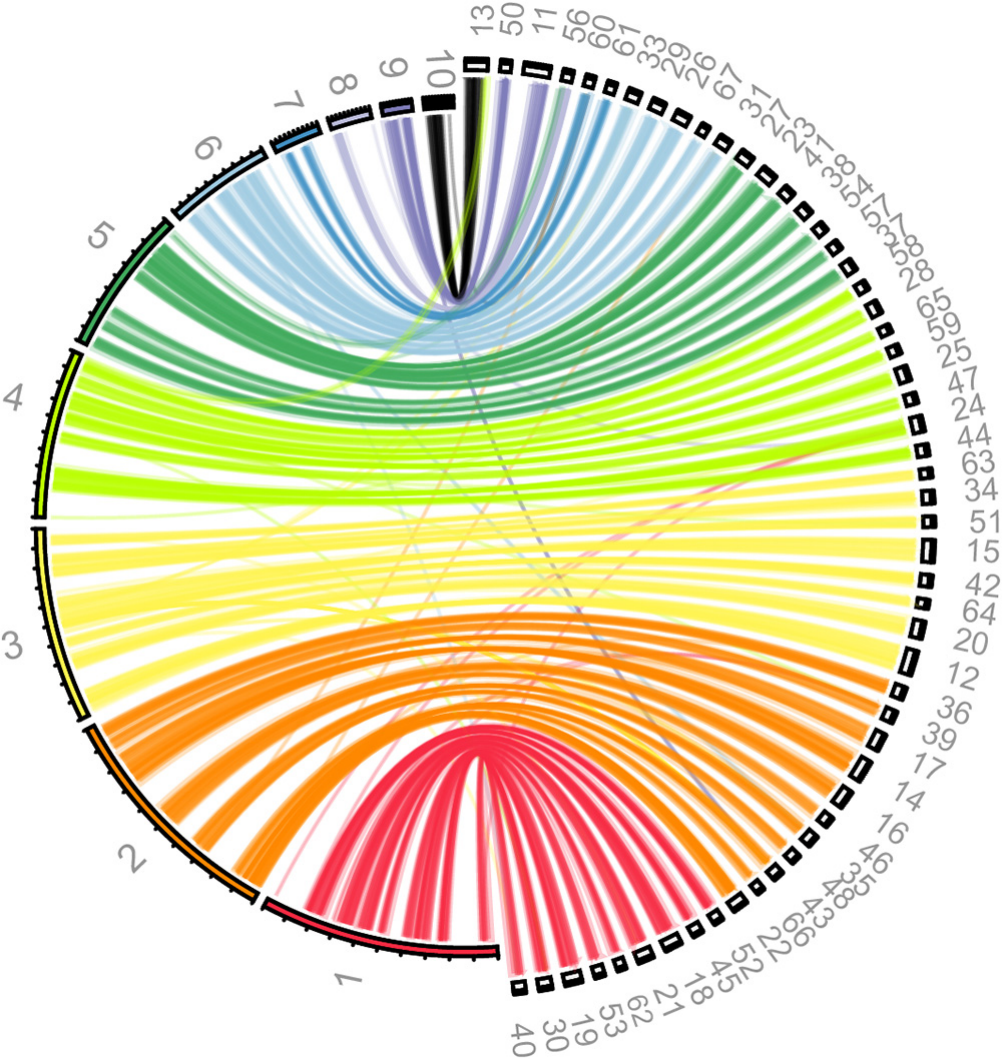
C. sanyaensis UCD423 has a diploid genome. Similarity between the two haplotypes of C. sanyaensis UCD423 was visualized with Circos (11) and Circoletto (12) as described by O’Brien et al. (13), using the diploid assembly after polishing with Pilon. The 10 scaffolds from haplotype A are shown on the inner ring on the left. Scaffolds of  ≥100 kb from haplotype B are shown on the outer ring on the right. Smaller scaffolds are omitted for clarity. Sequences with similarity were identified by BLASTN, and alignments are plotted as links between the two haplotypes. The matches are colored with respect to the scaffold in the first haplotype. Scaffold 8 contains the ribosomal DNA (rDNA) and has few matches with scaffolds from the second haplotype outside the rDNA region, even when scaffolds of <100 kb are included. Assembly of the two haplotypes might have collapsed around the rDNA locus, possibly because of loss of heterozygosity. Matches to scaffold 56 suggest that scaffold 5 and scaffold 8 should be joined. Similarly, matches to scaffold 13 suggest that scaffold 4 and scaffold 10 should be joined. The total length of a single haplotype is approximately 15.4 Mb. The average sequence identity between the haplotypes is 96.72%, as calculated from scaffolds of >100 kb using the average nucleotide identity (ANI) calculator described by Rodriguez and Konstantinidis (14) with default parameters.

Using the diploid parameters with Canu resulted in an assembly that largely kept two haplotypes separate (Fig. 1). The first haplotype (haplotype A) is represented by the largest 10 scaffolds, ranging from 2.94 Mb to 369 kb (Fig. 1). The second haplotype (haplotype B) is fragmented into much smaller contigs, each of which matches a contig from the first haplotype (Fig. 1). Therefore, C. sanyaensis has a diploid genome and the haplotypes differ by ∼3.3%, suggesting that the genome results from hybridization between related but not identical parents (9). The final diploid assembly of the MinION data was error corrected by incorporating the Illumina data, using nine rounds with Pilon v1.23 (10).

### Data availability.

This whole-genome shotgun project has been deposited in DDBJ/ENA/GenBank under accession number CAJVQF00000000. The raw reads from Illumina sequencing are available under SRA accession number ERR6313261 and those from MinION sequencing under SRA accession number ERR6310792. These data are also available under project PRJEB46370. The ITS sequence is at accession number MZ507576.
